# Unorthodoxy in Medicine and Religion
*Presidential Address to the Bristol Medico-Chirurgical Society, 10th October 1962.


**Published:** 1963-01

**Authors:** A. M. Maclachlan


### UNORTHODOXY IN MEDICINE AND RELIGION*

BY

A. M. MACLACHLAN

### I. JOHN WESLEY

The eighteenth century was the age of enlightenment. In that part of it which concerns us this evening between 1740 and 1780, we find a generation of men self-poised, self-approved, freed from the disturbing passions of the past and not yet troubled by the different world which would soon be upon them as a result of the Industrial and the French Revolutions. It was a moment of peace between the religious fanaticisms of the past and the fanaticisms of class and race which were to follow. It was in the words of Trevelyan the age of aristocracy and liberty; of the rule of law and the absence of reform; the age of unchallenged assumptions when philosophers had ample leisure to moralise on the human scene, in the happy belief that the present state of society was permanent. It was the classical age, the final outcome of reason and experience: it regarded itself not as setting out but as having arrived. The medieval period had sunk below the horizon and men looked back with a sense of kinship to the ancient world. The upper classes regarded the Greeks and Romans as honorary Englishmen, their precursors in liberty and culture, and the Roman Senate as the prototype of the British Parliament. It was the best of all ages to live in if you had influence and wealth.

But for the less privileged, the labourer, the Kingswood miner and the peasant poor it was far from being Merrie England. For them it was an age of ignorance and brutality; of dirt and squalor, of disease-ridden prisons, of overcrowding in small insanitary dwellings, of open sewers and uncontrolled epidemics, of a death rate which would appal us to-day. J. H. Plumb writes in "*England in the XVIII Century*": "The first noticeable thing about Bristol would have been the stench. There was no sanitary system and the poor made a public convenience of every nook and cranny. The unpaved streets were narrow: many of the Bristol streets were too narrow for carts, and sledges had to be used for moving goods. Most cellars were inhabited not only by people but also their pigs, fowls, sometimes even by their horses and cattle. All tradesmen and craftsmen used the street as their dustbin, including butchers who threw out the refuse of their shambles to decay and moulder in the streets". Hospitals were a poor remedy for the sick poor and in many cases were appalling places for those who were obliged to go to them. The four-poster bed and the straw mattresses on which the sick were nursed, the fear of draughts and a general ignorance of the causes of infection, made them breeding grounds for disease rather than curative institutions.

The number of doctors was thinly spread; there were not enough to go round. Four per year graduated from Oxford, five from Cambridge and about sixteen per year from Edinburgh. In addition there were the guilds of apothecaries and the barber surgeons; these provided an inferior type of practice but were confined for the most part to the larger centres of population. England in the eighteenth century was a sparsely populated land with only a few large cities. The total population at the beginning of the century was only $4\frac{1}{2}$ millions. It was a mainly rural community and over the major part of the country inaccessibility and the poverty of the people made it impossible for a regular physician to earn a living. Besides, the general standard of practice of medicine was exceedingly low. The whole of medicine was considered

* Presidential Address to the Bristol Medico-Chirurgical Society, 10th October 1962.

to be within the compass of a well educated gentleman. Many parish ministers were physicians as well as priests. Comrie in his "History of Scottish Medicine" says that the people in the outlying districts depended on the landowner or the clergy for all the medical ministrations they needed: in England the poor people looked to the Squire or the Vicar. Richard Baxter was not only Parish Minister of Kidderminster but the local and very successful physician as well. Crabbe is mostly remembered as a poet; we forget he was clergyman and doctor as well. Bishop Berkeley the philosopher was one of the most successful physicians of his time, although he is now best remembered as a philosopher and philanthropist. It was he who made Tar Water one of the most popular remedies of the eighteenth century.

In London the physician seldom saw the patient. The great man, dressed like a gentleman of his day, his French rapier dangling by his side, repaired to the coffee house where he was interviewed by the men of lower medical and social status than the physician. There he would be consulted by the apothecary who would describe the case to him, whereupon the physician would write a prescription properly couched in Latin for administration to the patient. It was rare for the great man actually to see the patient: rarely had he any real contact with the sick.

Judged by modern standards 18th century standards of medicine were appallingly low. There were a few enlightened physicians, but the majority were prejudiced in their approach to the subject of medicine and the healing art by the philosophical conceits of their day. Anatomical knowledge was superficial, physiology was an uncharted field, pathology was unknown and illness had to conform to certain types and these types determined treatment with often disastrous results. According to those doctor-philosophers of the day there were four doctrines about medicine—of similars, of signatures, of analogy and contagion. Thus the doctrine of similars indicated that yellow birds properly reduced to physic would be curative for jaundice, or that stewed raven would prevent black hair from turning grey. The doctrine of similars which really dates from the sixteenth century taught that medicines composed from plants or animals whose markings or shape suggest the organ to be treated will be effective in the treatment of such organs. Thus cyclamen having the shape, or in the phrase of the time, the signature of the ear, will be effective in the treatment of conditions associated with the ear. Similarly the liver-like shape of the leaf of the liver wart will be effective in all diseases connected with the liver. In the doctrine of analogy cures were deduced from the behaviour of ailing animals. This was perhaps the least dangerous of all the doctrines; on the other hand the doctrine of contagion had little to commend it. It found cures in things imagined to be associated with the distemper. It thus imagined that there was an association between the phases of the moon and lunacy and therefore recommended moonstone in the treatment of mental illness.

It is against this background—a background of a sparsely populated England with primitive communications; of ignorance, poverty and squalor; of medicine in the hands of the coffee house gentleman dandy; of the ill educated apothecary or barber surgeon; and of medicine corrupted by philosophical conceits that I want to introduce John Wesley in his less known role as an unorthodox physician of the eighteenth century.

There are two conflicting views about Wesley. The first is that he was a malign influence in a socially changing England; that at a time when Scotland as well as England was producing an astonishing galaxy of talent, poets, philosophers, men of letters, scientists, engineers, architects, Wesley and his preaching laid a stifling hand on England making men think of their sin rather than their misfortune, and of escaping the fires of hell rather than of breaking the chains of oppression and poverty which bound them to their past. It is claimed that this unnatural obsession with sin and hell paved the way for much of the religious hypocrisy of the nineteenth century with its emphasis on personal salvation rather than on social justice. On the other hand it is claimed that Wesley may have saved England from bloody revolution and an English Bastille; that his teaching had social overtones, turning men from drunkards into sober and industrious citizens determined to improve society by example rather than revolution; that social progress since Wesley has been by parliamentary means and the ballot box and never by blood and the sword; that socialists in England, who are the political sons of the first Methodists, have never been Marxists—in a word that by the twentieth century we had had a social revolution as significant and far reaching as any in our history, without a drop of blood being shed; all this is due in part to Wesley who showed us the more excellent way.

In 1746 Wesley opened his first dispensary in Bristol and in 1747 he published the first edition of his book *Primitive Physic*, or an *Easy and Natural Method of curing Most Diseases*. Since "Primitive Physic" tells us all we need know about Wesley as a doctor for the purpose of this lecture I will let him speak for himself. He believed that cures are not to be found in philosphy but by accident and proved by experience. "It is probable" he says "that physic as well as religion was in the first ages, chiefly traditional, every father delivering down to his sons what he had in like manner received, concerning the manner of healing and the medicines which were of the greatest efficacy for the cure of each disorder". This is what Wesley means by "Primitive Physic". His declared method was a "plain and easy way of curing most diseases". "I have only consulted" he says "experience, common sense and the common interest of mankind." He would have none of the learned polypharmacy of the fashionable physicians of his day. "As soon as you know the distemper first use the first of the remedies for that disease which occurs in the ensuing collection. Secondly after a competent time if it takes no effect use the second, the third and so on. And supposing" he adds "men can be cured this easy way who would desire to use any other; who would not wish to have a physician always in his house and one who attends without fee or reward." "Observe" he continues "all the time the greatest exactness in your manner of living; abstain from all mixed, all high seasoned food. Use plain diet, easy of digestion and this as sparingly as you can, consistent with ease and strength. Drink only water if it agrees with your stomach; if not good clear small beer. Use as much exercise daily in the open air as you can without weariness. Sup at 6 or 7 on the lightest food, go to bed early and rise betimes. To persevere with steadiness on this course, is often more than half the cure."

"The great rule of eating and drinking is to suit the quality and quantity of our food to the strength of our digestion."

"Nothing conduces more to health than abstinence and plain food with due labour."

"Tender persons should have light suppers, and that two or three hours before going to bed."

"A degree of exercise is indispensably necessary to health and long life. Walking is the best exercise and the open air when the weather is fair contributes much to the benefit of the exercise."

"The studious ought to have stated times for exercise. The fewer clothes anyone uses by day or night the hardier he will be. Cold bathing is of great advantage to health; it prevents abundance of diseases. It promotes perspiration, helps the circulation of the blood and prevents the danger of catching cold."

In some things Wesley was away ahead of his time. Listen to this: "I recommend that every baby be weaned at 7 months"; and this in an age when it was usual to keep a child at the breast for 18 months to 2 years. Or this: "for one seemingly killed with lightning or drowned, blow strongly with bellows down his throat; this may recover a person seemingly drowned. It is still better if a strong man blows down his mouth". What could be more modern than that in the way of treatment? When Wesley's first edition of "Primitive Physic" was published the cause of scurvy was unknown; "Live on turnips for a month" is his recommendation. In the 1780 edition he advises "Two Seville oranges a day, or morning and evening a spoonful or two of lemon juice and sugar". "It is" he says, "a precious remedy and well tried". It is significant that in all editions of his book a preponderance of his remedies has to do with fresh vegetables. The latest novelty of the second half of the eighteenth century was electricity. Wesley was one of the few who thought it would have any significance in medicine. He strongly recommended severe electric shock in the treatment of hysteria. Not quite right—but for the eighteenth century a brilliant near miss.

There are over 800 suggested remedies in "Primitive Physic" but I am bound to say that not all are up to the standard of those I have selected, and it is easy to ridicule Wesley for some of his cures. But had orthodox medicine anything better to offer? When he opened his dispensary in Bristol there was already another free dispensary at the Royal Infirmary: how much more enlightened were its physicians than Wesley? One of the Infirmary physicians was Dr. John Paul, a great advocate of phlebotomy. One of the Surgeons, a Mr. Metford, has left on record that on one day he bled 30 patients on the advice of Dr. Paul. Once, being present when Dr. Paul was seeing patients in the Admission Room, he observed that Dr. Paul's first question to the patient was "Are you a Bristol man?" If the answer was in the affirmative Dr. Paul at once ordered a venesection and the withdrawal of 20 oz. of blood. When Mr. Metford ventured to ask the reason for this procedure Dr. Paul replied: "Because Sir, if he is a Bristol man he sits of an evening smoking tobacco and drinking fat ale: the first thing to be done therefore is to let some of that run out and then we shall see what else is the matter".

Dr. Paul would order bleeding for a pleurisy. John Wesley's comment on this is "no end is answered by bleeding in a pleurisy which may be much better answered without it. I would advise the application of a brimstone plaster, for to what end should a patient lose 20 oz. of blood just to oblige the doctor and the apothecary". If I have to choose in the treatment of pleurisy between Dr. John Paul and Rev. John Wesley I confess I am a Wesleyan.

### II. MARY BAKER EDDY

And now we move on 100 years to America and the nineteenth century. In the year 1866 Mary Baker Eddy had just had a revelation which was the starting point of Christian Science. Not that Mary Baker Eddy's revelation was wholly original. The idea of a benevolent God which is central to Christian Science had developed as New England Puritanism had gradually mellowed into universalism and unitarianism. The United States had bade farewell to the Europe of the eighteenth century; this was no longer the age of enlightenment, of gracious living, of Mozart, Hume, Dr. Johnson and Voltaire, this was the New World which owed nothing to the old. It was the age of optimism, of progess; the age of opportunity unfettered by the chains of the Old World. The time was ripe; new bottles were needed to hold this new heady wine. Charles Darwin had published his *Origin of Species* seven years before and Darwin's findings matched the American mood. Science reflected the dominant ethos of nineteenth century America which had grown accustomed to the seemingly limitless expansion of the frontier further and further west. A new religion was required to match the mood of this new thinking; one which would combine the business ethic, material progress and the new science in one great religious concept. This explains the phenomenal growth of Christian Science in the United States in the first few decades of its existence.

Mary Baker Eddy was born in 1821 in New Hampshire where she spent almost all of the first forty-five years of her life. For long periods she was a semi-invalid. Her religious background was the restricting atmosphere of New England Puritanism.

Long before 1866 she had been interested in various methods of healing. "I sought knowledge", she said, "from the different schools: allopathy, homoeopathy, electricity, hydropathy and from various humbugs but without receiving satisfaction." Before 1866 she had been associated with Phineas Quimby, a New England healer who after dabbling in mesmerism became convinced that all healing processes were purely mental, to be achieved by the attainment of certain attitudes of mind on the part of the patient. To Mrs. Eddy this was as seed falling into prepared ground, for there is little doubt that the revelation of 1866 came to a woman preoccupied with sickness, with healing, and with religion.

Following her revelation she began classes of instruction in her methods of healing, advertising in a spiritualist journal. Her charge was 100 dollars, later raised to 300 for twelve lessons; an illustration, I suppose, of what nineteenth century liberals called the law of supply and demand. From these instruction classes grew the Christian Science Association and in 1879 the First Church of Christ Scientist was founded in Boston. Mrs. Eddy was now well launched and on her way. In 1897 she sent one of her disciples, Julia Field-King, to England, and she soon had a distinguished following in London including members of the Peerage and Sir Douglas Galton F.R.S. a former President of the British Association. In a short time the 1st Church was opened in London and by 1926 Christian Science had congregations in every state of the American Union and had grown more rapidly than any other denomination. By 1931 there were over 100 Churches in England and the Movement was firmly established in Germany, Switzerland and Holland.

And now let us consider the teaching of this Sect. The theology of Christian Science is Unitarian. God alone is worshipped, Jesus is the Way Shower and the Holy Ghost is Christian Science. Jesus is not unique and many followers of Christian Science regard Mrs. Eddy as at least his equal. Man is made in the image of God and because God is spiritual, so must man be. Christian Science holds that in the absolute sense man is spiritual and therefore cannot be sick. Man thinks of himself as material and subject to death and decay. This is an illusion but an illusion so powerful that it causes man to feel and suffer as if it were so. In point of fact there is no sickness to heal except a false belief which man holds of himself. Treatment is a process of affirmation and denial. The practitioner begins by allaying the fear of the patient. "If you succeed in wholly removing the fear, your patient is healed" wrote Mrs. Eddy. The practitioner then proceeds with a reaffirmation of God's allness and goodness. "Realize the presence of health and the fact of harmonious being until the body corresponds with the normal conditions of health and harmony". The practitioner will go on to argue that since God is Spirit and man reflects God he cannot be sick. Sickness is but a dream from which the patient must be wakened. In practice, however, the system is equivocal and in spite of their claim to heal disease without medical aid (Mrs. Eddy claimed that Christian Science cured more cases of cancer than all the doctors) they do from time to time resort to orthodox practitioners. Mrs. Eddy herself went so far as to say "Until the advancing age admits the efficacy and supremacy of Mind it is better to leave surgery and the adjustment of broken bones and dislocations to the fingers of the Surgeon". Ambivalence is a weakness of the Sect. Practitioners are advised not to handle infectious or contagious diseases which are notifiable under State Health regulations, and in the Second World War the Christian Science Directors found it necessary to issue a directive that when Army and Navy regulations made it necessary a Christian Scientist could submit to orthodox medical or dental treatment or treatment in Hospital. This conforms to a previous directive from Mrs. Eddy: "Rather than quarrel over vaccination", she said, "I recommend, if the law demands, that an individual submits to this process and then appeal to the Gospel to save him from any bad physical results". Christian Scientists pay regular visits to their dentist and do not cavil at wearing spectacles.

It is clear that they effect numerous compromises between religion and the demands of the material world in the matter of healing. I have had devoted Christian Scientists who in the end have been glad to resort to orthodox medicines for organic disease.

And now a brief glance at the social structure of this Society. In Great Britain it is almost entirely an urban phenomenon. It is in the suburbs and dormitory areas that it has found its most congenial atmosphere, and even more particularly in the spas and watering places. It is estimated that in England it is more than 99 per cent urban. It seems to flourish best in fashionable watering places. Quite apart from the social class of those who can afford to visit or reside at seaside resorts such towns naturally draw to themselves numbers of ailing and afflicted persons and many who have not only money but a morbid preoccupation with their bodily health. Such people are clearly a ready-made recruiting ground for Christian Science. It is significant that in the larger urban centres with predominantly working class populations it gains no foothold. Manual workers, factory workers, the poor and uneducated are not attracted; its votaries are principally drawn from the middle classes; it prospers best where there prevails a fair standard of comfort; where economic pressure is great and there is little time or facility for speculation the cult does not flourish. Of the 233 Christian Science practitioners whose addresses are listed in London not one is resident in the East End, but they flourish in Kensington, Westminster, Chelsea, St. John's Wood and Mayfair. Of all Christian Science practitioners in the world to-day 77 per cent are married women, and married and single women taken together account for 88 per cent of the total number of practitioners. Patients are drawn from a very narrow field; usually they are the converted or the desperate, those who have lost faith in the medical profession or those abandoned as incurable. There are few young people among them and the elderly predominate. It is also significant that the Faith has spread only in Western Countries and those centres of Asia and Africa where Western culture is at its strongest. It undertakes missionary work in the Protestant World where there is a high standard of living but makes no attempt to convert the non-Christian or preliterate peoples of either Asia or Africa. It has made no progress in Catholic countries nor has it penetrated strongly Catholic areas in Britain or America. It is not a family religion; not an inherited belief but rather an adopted cult with an essentially individual appeal. It is too esoteric to meet the needs of the ordinary man; it has none of the panache or the robust common sense of Wesley; and finally it is significant that since the passing of the National Health Act, Christian Science has ceased to be a growing movement in this country.

### III. RUDOLF STEINER

My third example of unorthodoxy comes from Germany—Germany in the early 1920's with its political and economic confusion. For 4 years Germans believed they were winning the War: only for a month were they faced with the truth of defeat. There followed a period of galloping inflation which brought unemployment to the poor, while the savings of the middle classes vanished betwixt dawn and dusk. The same background which produced Adolf Hitler produced Dr. Rudolf Steiner—the need for an explanation for the chaos in Germany and for the voice which would restore the shattered confidence. Like Hitler, Rudolf Steiner was an Austrian but of an earlier generation. His father was a station master employed by the South Austrian Railway. From school he went to the Technical University in Vienna to study natural history, chemistry and mathematics. On graduating he was invited to edit the scientific works of Goethe, for a new edition which was soon to be published. Recognised as the greatest of German poets, Goethe's achievements in other fields are less well known, and the least well known are his excursions into the field of science, though he himself thought his scientific and philosophical studies more highly to be prized than his poetry. Steiner's studies became as discursive as Goethe's, ranging from philosophy and metaphysics to architecture and biology. Following years of study at Weimar and Berlin, Steiner took to lecturing on what he claimed to be "spiritual science" as opposed to "natural science" and based on the work of Goethe, finding his main audience among members of the Theosophical Society. Just before the outbreak of the First World War Steiner broke with the Theosophical Movement which was not Western enough in its conceptions of spiritual phenomena for his Teutonic mind; and this led at the close of the War to the founding of the New Society with which his name is closely associated, the Anthroposophical Society.

My interest in Rudolf Steiner began last year when I was invited to join the Council of St. Christopher's School for handicapped children on Durdham Downs founded some years ago by two ladies, Miss Groves and Miss Grace, both of whom were members of the Anthroposophical Society, the School being run on educational lines laid down by Rudolf Steiner. As 1961 was also the centenary of Steiner's birth with exhibitions by the Society in many of the large cities of England—including one in Bristol Corn Exchange—literature about the man and the Society were the more easily come by.

But to return to the Germany of the Versailles Treaty. Just as Hitler sought a political explanation and solution to the problem of a humiliated and chaotic Germany, so Steiner sought a scientific and religious one. It all began, said Steiner, with Isaac Newton and the birth of Natural Science. For although Robert Boyle, Isaac Newton, and the early members of the Royal Society were religious men who repudiated the sceptical doctrines of Hobbes they familiarised men's minds with scientific methods of inquiry to discover truth. And although Newton lived and died in the orthodox faith of a miraculous and revealed religion his law of universal gravitation and his Calculus supplied methods of approaching truth that had no relation to theology. Trevelyan in his "English Social History" tells us that the Bishop of Rochester who wrote the first History of the Royal Society, commends the learned and inquisitive age in which he lives, praises the practical objects of the Royal Society and claims for these new philosophers the widest range of inquiry, these two subjects God and the Soul being only forborne—in all else they wander at their pleasure. No attempt was now to be made to fit the findings of science into the scheme of theology as the schoolmen of old had striven so long and so painfully to do. God and the Soul were to be taken for granted—and left aside. By the twentieth century science had replaced religion and art as the driving force in human life. The main stream of life, said Steiner, is scientific and technical; on its practical side it is economic, industrial and commercial. Life is focussed on the physical senses and the material world. Religion and art are tolerated, they are valued, but they do not enter into everyday life as they did in past civilisations, in ancient India or Egypt or Palestine or Greece or in Mediaeval Europe. Modern life is no longer religious but economic. Religion has been ousted from the sphere of cosmologic wisdom and restricted to the sphere of personal morality and social ethics. Science has created a world where man does not belong. If man is in any sense religious it is an added extra to life, for those who happen to like it and have time for it. The religious man of the twentieth century is a spiritual epicurean. Yet the result is an even greater abyss between man's thinking and man's doing. How could he, in an age when the logic of science was supreme, behave in such an unscientific way? Where was the scientific logic of the Somme, Paschendaele, the blockade of Germany and the hundreds of thousands of children dying of malnutrition in Europe? Why this gulf between human knowing and human doing? At a time when humanity was equipped as never before to investigate and proft from the order of the Universe, human life had fallen into utter chaos.

Man, said Steiner, must return to the cosmologic wisdom of religion. Not the religion of a pre-Newtonian age but one which would be scientific and meet modern thinking. The answer is anthroposophy—man-wisdom or "spiritual science" as opposed to "natural science". Steiner accepted the challenge which the schoolmen of Newton's day declined: the dualism between science and religion could be bridged. Steiner would have echoed the words of Plato. "This is the greatest error in the treatment of sickness", wrote Plato two thousand years ago "that there are physicians for the body and physicians for the soul, and yet the two are one and indivisible." Anthroposophy is but knowledge of the true being of man and of his relation to the universe. Eddington is quoted as saying that "all our knowledge of the universe could have been reached by visual sensation alone" and Steiner's comment on this is that, in order to obtain scientific cognition of the physical world, man has felt constrained to surrender the use of all his senses except the sense of sight. Science is concerned only with the world of physical sense perception. But behind the physical realities which we perceive there are super-sensible realities. Steiner claimed he had direct personal perception of these super-sensible realities and that at a future stage of evolution it would be a universal faculty of mankind. He claimed that man had a spiritual and not a physical origin and that he had failed because of his materialistic world outlook produced by natural science which had impeded the true course of spiritual evolution. The spirit world is the real world—not "other than" or future to the physical world—it is the physical world. Man does not start his existence at birth or finish it at death; his spirit exists before birth and after death. It is difficult to follow Steiner in much that he wrote, for while he strove to give his philosophy a modern dress and clothe it in scientific jargon, much of it is mediaeval mysticism together with what he learned through long contact with theosophy and the religions of the East. At any rate he did not in his lifetime meet with much success in his own country. Dismissed by Annie Besant from the Theosophical Society, his Spiritual Science was not to the liking of the Germans licking their wounds after the 1st World War. He soon was in conflict with Hitler's National Socialist Party; his lectures were broken up, his life threatened, and he had to seek refuge in Switzerland where he remained till his death in 1925.

After his death however, this philosophical curiosity was taken up by others both on the Continent and in this country; for, though Steiner had a technical and not a classical education, the colossal arrogance of the system extended to every sphere of life—to politics, education, medicine, agriculture and architecture. I would not bore you with these details but for the fact that anthroposophy remains in this country as a specialised system of education for handicapped children, and there are still those who hold Steiner's curious views on physiology and the treatment and cure of the sick. The approach to the handicapped child at St. Christopher's School in Bristol—cretins, mongols, cases of hydrocephalus and so on—is that since man is spiritual and not physical the human spirit can never be ill and mental defect is a sign or symptom of physical handicap only. If the spiritual being unites itself with a perfect body all is well: but if the physical instrument through which the spiritual being operates (say the brain of a child) is injured, then the damaged instrument prevents the spiritual from fully expressing itself. Hence curative education in which doctor and teacher must collaborate to overcome as far as possible the physical handicap which makes the child an imperfect being. By "doctor" those who follow Steiner's methods do not mean an orthodox medical practitioner, but one with a medical qualification who is also trained in anthroposophical methods and who accepts Steiner's teaching in physiology and therapeutics. This teaching Steiner elaborated in a series of lectures which he gave at Dornach in Switzerland in 1920. There are, he said, three principles at work in man, thinking, feeling and willing, and these three activities are related to different parts of the body. There is the head system, i.e. the brain and nerves, which is the seat of thinking. Then there is the rhythmic system which include the functions of breathing and the circulation of the blood and all other functions which express themselves in the rhythmic processes of the body. Being chiefly situated in the thorax the second system is called the Chest System. The Chest System is concerned with feeling and feeling is based on the rhythmic processes of breathing and circulation. The third system is the lower or digestive system which is assimilatory and is concerned with will.

On this unorthodox system of physiology is based an even more absurd system of therapeutics. Let me give you an example. Here is a case history from the Clinic of Dr. Ita Wegman at Dornach. "A woman patient 26 years old, came to our Clinic suffering from the serious consequences of influenza and catarrhal pneumonia which she had undergone in 1918. This had been preceded in 1917 by pleurisy. Since the influenza she had never properly recovered. In 1920 she was very much emaciated and in a feeble condition, with slight temperature and nocturnal perspiration. Soon after the influenza she had begun to suffer from pains in the back which grew increasingly till the end of 1920. Then, with a violent increase in the pain, curvature of the spine became apparent in the lumbar region. When the patient came to us, she was suffering from a gravitation abscess on the right thigh. Her body was distended and she had slight ascites. There were catarrhal noises over the apices of both lungs. Spiritual-scientific investigation revealed a hypersensitiveness of the astral body and the Ego-organisation. Now the astral functions are disintegrative. Thus the general vitality and the normal process in the physical organs were necessarily atrophied. Such a condition is always accompanied by processes to some extent extra-human, taking place within the human organism. Hence the gravitation abscess, the lumbar pains, the distended abdomen, the catarrhal symptoms in the lungs. The therapeutic treatment must therefore seek to lower the sensitiveness of the astral body and the Ego-organisation. This may be done by administering silica, which always strengthens the active inherent forces against undue sensitiveness. We also gave silver injections at a high potency and the fever declined. The patient left the Clinic with a twenty pounds increase in weight, and considerably stronger."

I think you will agree that what is described here is a clear case of advanced general tuberculosis with a psoas abscess in spite of all the jargon about astral bodies. I am glad to be able to assure you that at St. Christopher's School, medical care of the sick child is in the hands of a competent orthodox medical practitioner, who is a member of this Society. The role of the anthroposophical doctor is to collaborate with the teacher in the field of education only.

Here I would like to digress for a moment and, if I may, give you a personal reminiscence. Those of you who are not of my generation, who have no memories of the 1920's following the upheaval of the 1st World War, when radicalism as a political faith for the young gave way to socialism, cannot understand how much our thinking was influenced by George Bernard Shaw. Here was a political prophet in our eyes worthy of the new age in which we lived. We were carried along by the magic of his pellucid prose and in a trice some of us were halfway to Fabianism. Then having avidly devoured Shaw on politics and economics, subjects upon which I was largely an innocent, I proceeded to read him on Religion and Medicine, (I was a medical student at the time), two subjects about which I had at least some knowledge. To my amazement I found Shaw on both subjects could write the most arrant nonsense. One still admired the prose, the syllogisms were cleverly contrived, but this was not a prophet but a jester carefully arranging his Aunt Sallies and then knocking them down with the brilliance of his dialectic. And I concluded that if Shaw could write nonsense on the two subjects about which I knew something, in all probability he equally wrote much that was nonsense on the other subjects where, in my ignorance, I had taken him as my guide. And so I decided to go back to square one and find a more reliable political guide.

I mention that experience for it recurred last year when I tried to find my way through the labyrinth of the philosophical and metaphysical works of Rudolf Steiner. If he could write such nonsense as I have indicated to you about physiology and therapeutics one may be justified in the assumption that the leopard does not change his spots, even when he moves into another field.

Ladies and Gentlemen, I expect you have had enough. Steiner developed most of his philosophical and scientific ideas from the works of Goethe. Goethe remains a great poet but an indifferent scientist; and with that verdict we can leave Rudolf Steiner to rest in peace. By all accounts he was a good man, and as Pascal says "the heart also has its reasons"; one can admire the heart even if one deplores the head.

One final word. A lecture unlike a sermon need not point a moral, but if you feel that this lecture has so many of the qualities of a sermon that a moral is indicated I can only direct you to the well worn cliché that "we live in a queer world". And in the field of medicine and religion, as they say in Yorkshire, "There's nowt so queer as folk".

